# Functional Identification of Target by Expression Proteomics (FITExP) reveals protein targets and highlights mechanisms of action of small molecule drugs

**DOI:** 10.1038/srep11176

**Published:** 2015-06-08

**Authors:** Alexey Chernobrovkin, Consuelo Marin-Vicente, Neus Visa, Roman A. Zubarev

**Affiliations:** 1 Division of Physiological Chemistry I, Department of Medical Biochemistry and Biophysics, Karolinska Institutet, Scheelesväg 2, SE-17 177 Stockholm, Sweden; 2 Department of Molecular Biosciences, The Wenner-Gren Institute, Stockholm University, Stockholm, Sweden

## Abstract

Phenomenological screening of small molecule libraries for anticancer activity yields potentially interesting candidate molecules, with a bottleneck in the determination of drug targets and the mechanism of anticancer action. We have found that, for the protein target of a small-molecule drug, the abundance change in late apoptosis is exceptional compared to the expectations based on the abundances of co-regulated proteins. Based on this finding, a novel method to drug target deconvolution is proposed. In a proof of principle experiment, the method yielded known targets of several common anticancer agents among a few (often, just one) likely candidates identified in an unbiased way from cellular proteome comprising more than 4,000 proteins. A validation experiment with a different set of cells and drugs confirmed the findings. As an additional benefit, mapping most specifically regulated proteins on known protein networks highlighted the mechanism of drug action. The new method, if proven to be general, can significantly shorten drug target identification, and thus facilitate the emergence of novel anticancer treatments.

Target-based discovery is the way pharmaceutical industry most often uses in searching for new drugs, with compound libraries screened for binding or activity against a known protein target. In contrast, phenomenological screening of small molecule libraries is a “black-box”, target-agnostic approach, where compounds are interrogated in cell-based assays with a readout linked to a disease-relevant process (e.g., cancer cell apoptosis). Arguably, this latter approach to drug discovery offers better chances for success. This is because the assay is more relevant to human physiology, and a multitude of targets are addressed simultaneously. Indeed, between 1999 and 2008, of the first-in-class compounds that were approved by the FDA, almost two thirds had been derived from phenotypic screening^1^. However efficient, this approach has a serious bottleneck - drug target discovery and validation. Less than 200 small-molecule anticancer drugs approved by FDA have a known mechanism of action, while many thousands of promising molecules remain with poorly characterized or completely unknown targets^2^,^3^. This mismatch between the multitude of promising compounds and the limited knowledge of the targets and underlying mechanisms of action represents one of the greatest unmet needs in the war against cancer.

Strong demand for novel unbiased methods of drug target identification has been stressed in a recent review by Schenone *et al.*^4^. It has been demonstrated that omics-based methods have become highly valuable in characterization of targets of small molecule drugs. Mass-spectrometry based proteomics is a well established tool in drug discovery^5^. Unlike transcriptomics that covers the whole range of expressed genes, a typical untargeted 1D LC-MS/MS proteomics experiment can usually detect and quantify up to 5,000 proteins, which is less than half of the expressed human proteome^6^. However, proteomics technology rapidly progresses, and deep proteome analysis with more than 8,000 protein groups quantified is becoming increasingly available^7^,^8^,^9^,^10^. Besides, measuring relative protein concentrations accounts not only for protein expression but also for protein degradation, which makes proteomics particularly valuable in drug target discovery.

Despite substantial recent developments, such as chemical proteomics^11^, dynamic proteomics^12^, and the use of pathway analysis^13^, unbiased drug target discovery remains a significant challenge. Most recent success has been achieved by Savitski et al. who combined cellular thermal shift assay (CETSA) with quantitative mass-spectrometry for extensive characterization of several kinase inhibitors^14^. Unbiased measure of drug-target occupancy has been obtained for multiple targets. However, the list of revealed potential drug target candidates has been relatively long, and not all true targets could be found^14^. Thus, an alternative, general (or at least widely applicable) and more specific method that does not require chemical modification of the drug molecule, *a priori* knowledge of the drug action mechanism or signaling or metabolic pathways involved, would be highly valuable. Here we describe such a method that we call Functional Identification of Target by Expression Proteomics (FITExP). FITExP is based on our finding that, for the protein target of a small-molecule drug, the abundance change in late apoptosis is unexpectedly large compared to other proteins that are normally co-regulated with the drug target. In a proof-of-principle experiment described below, the method yielded known targets of several common anticancer agents among a few (often, just one) likely candidates identified in an unbiased way from >4000 proteins. A subsequent experiment with a different set of cells and drugs provided validation of the above findings. As an additional benefit of FITExP, mapping most specifically regulated proteins on known protein networks reveals the mechanism of drug action.

The brief history of insight that led to FITExP is the following. After studying the mechanism of action of the anticancer drug 5-FU by investigating the changes in the proteome of RKO cells treated with 5-FU^15^, it became apparent that the 5-FU target, protein TYMS, was significantly upregulated upon 5-FU treatment, especially in late apoptosis. The question emerged whether it was possible to “deduce” that the 5-FU target is TYMS, solely from the proteomics data, by sorting all proteins according to their regulation. Detailed data analysis showed that, even though TYMS was found among the most regulated proteins (top 5%), other molecules, in particular proteins involved in cell death, were regulated even stronger. This situation was similar to that found in other studies^12^,^13^. It became clear that, in order to deduce TYMS from the proteomics data, one needed to identify and “filter out” these generic cell death proteins. To do that, it was suggested to treat the cells with other drugs, and filter away the proteins that will always be found strongly regulated in apoptosis.

The follow-up experiment with several other drugs revealed that even that was insufficient to pinpoint TYMS as the most likely drug target. Then more specificity was added by employing, besides RKO, two more cell lines, under the assumption that the drug target should behave consistently, while unrelated proteins will be regulated in a cell-specific manner. Indeed, TYMS could now be identified as #1 or #2 most likely candidate among >4000 proteins. However, a new question arose – was the behavior of the drug target indeed a regulation or an extraordinary abundance change unrelated to regulation? If the former was true, then the behavior of TYMS in 5-FU treatment could be predicted based on the behavior of proteins that are co-regulated with TYMS in other treatments. After performing the corresponding analysis, it became clear that the change in the TYMS abundance in 5-FU treatment was extraordinary and unexpected. Finally, the same method of analysis applied to the data for other drugs clearly identified their targets as well, in a surprisingly strong agreement with existing knowledge. The last effort was to develop a statistical model to calculate the p-value of each protein to be the drug target, and to test the whole procedure in a second experiment with a different set of cell lines and drugs.

If proven to be general, the new method can significantly shorten drug target identification, and thus facilitate the emergence of novel anticancer treatments.

## Results

Details on the experiments and data analysis are given in Methods section. Here we provide only a brief overview of the experimental procedure and data analysis, focusing on the results.

### Proof of principle

In the proof-of-principle *Experiment A*, the objective was to test whether targets of common anticancer agents 5-FU, methotrexate (MTX), paclitaxel (PCTL), doxorubicin (DOXO) and tomudex (TDX) could be identified by expression proteomics. For that purpose, three cell lines (melanoma A375, lung cancer H1299 and colon cancer HCT116) were treated with drugs for 72 h, until deep apoptosis was reached and more than half cells were dead. As a control, untreated cells harvested at 0 h and 72 h, as well as cells starved for 10 days without media change (SEN), were used. All treatments and controls were grown in biological triplicates, which we believe is the minimal acceptable number of replicates in a proteomics experiment. From all samples, live cells were harvested, lysed, and the proteome extracted, digested and analyzed by LC-MS/MS. In total, 5,037 proteins were identified in 72 LC-MS/MS experiments with false discovery rate <1%. Label-free quantification across all 72 runs was performed for proteins that were identified with at least two unique peptides. The 4,168 proteins quantified in all treatments and controls were used for further analysis. FITExP analysis was made for 5-FU, MTX, PCTL and TDX, but not for DOXO that acts primarily via DNA intercalation.

In the validation *Experiment B,* we aimed to validate the findings of the *Experiment A* using smaller cell and drug panels. A different set of cell lines (colon cancer RKO and melanoma A375) was used, and the cells were treated with a different set of drugs: DOXO, 5-FU, PCTL and camptothecin (CAMP). After data processing, 3,570 proteins were quantified across all samples with at least two unique peptides. Tubulins that are known to be PCTL targets were not identified here because of their low abundances, and thus the FITExP analysis was made only for 5-FU and CAMP.

### Drug target regulation is unexpectedly large

**Figure 1:**
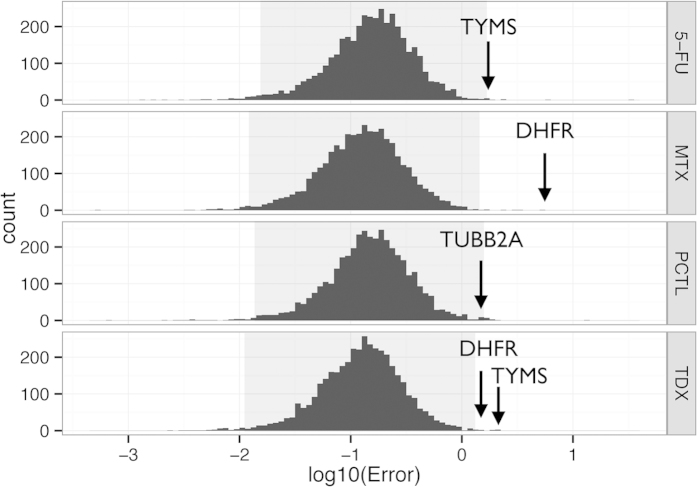
Distributions of the prediction errors of protein regulations based on co-regulated proteins reveal unexpectedly large regulation of drug targets (3σ area is shaded) .

In the dataset A, for each protein and each treatment we identified five proteins (“friends”) whose regulations *Reg* (relative abundance in a treatment normalized by the relative abundance in the untreated sample) in all *other* treatments and controls correlated most with that of the protein of interest. Linear regression of *Reg* values of “friends” gave a model for predicting the regulation of the protein of interest in a given treatment. Figure 1 shows that, for the treatments where the protein was the target, these predictions strongly (≥3σ) underestimated the experimentally observed regulations. Therefore, the targets’ regulations were not only strong, when a drug targeting them was applied, but they were unexpectedly strong. This observation created a basis for FITExP.

### FITExP approach

**Figure 2:**
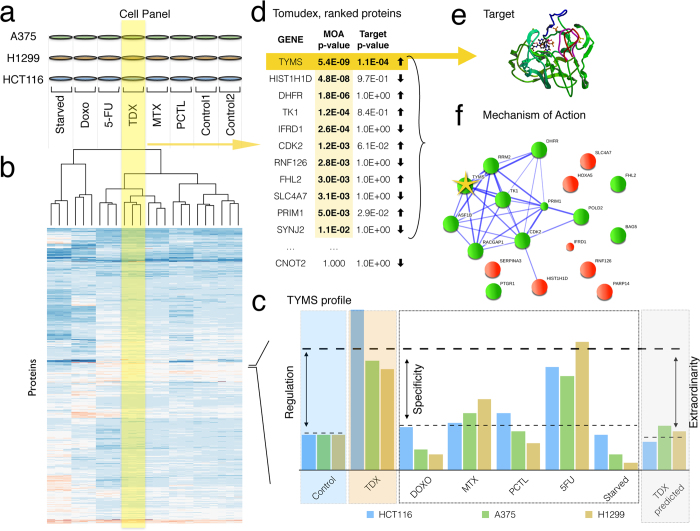
General workflow of the FITExP method of drug target identification: (**a**) a panel of cell lines is treated by a panel of drugs, in biological triplicates; (**b**) LC-MS/MS based proteomics identifies and quantifies ≥3,500 proteins, proteomic profiles are shown in a schematic heatmap with color-coded normalized abundances; the dendrogram shows hierarchical clustering of proteomic profiles with correlation-based distances; (**c**) for each protein, cell line and treatment, regulation *Reg*, specificity *Spec* and exceptionality *Exc* are calculated; (**d**) for each treatment, final protein ranks based on *Reg* and *Exc* are established and the p-values are calculated using Bonferroni correction; protein list is sorted in ascending order of p-values; (**e**) few proteins with p ≤ 0.05 (threshold p-value) represent the most likely drug targets; (**f)** top *n* proteins with p ≤ 0.05 according to *Reg* and *Spec* rankings are mapped on protein networks to identify the drug target mechanism.

The method’s workflow is depicted in Fig. 2. For every cell line, protein and drug treatment, three characteristics were calculated. Besides the regulation *Reg,* specificity *Spec* was defined as *Reg* for a given treatment normalized by the average *Reg* in other treatments and controls. The third value was the exceptionality *Exc*, which quantitatively assessed the unexpected character of protein’s regulation in a given treatment. *Reg, Spec* and *Exc* values were then subjected to rank product analysis that calculated the final ranks and p-values. For drug target identification, *Exc* and *Reg* gave an optimal combination that produces a short list of statistically significant candidates, while for mechanism of action, *Reg* and *Spec* provided a longer list of implicated proteins to be mapped on protein-protein networks. In each case, the resultant protein list was sorted by p-values in ascending order.

### Drug target deconvolution

Since only a few protein candidates were expected to be the targets of a particular drug in our experiments, Bonferroni correction was applied which ensures low risk (≤p) of even a single incorrect answer, and then the significant proteins (p < 0.05) were considered. Table 1 summarizes the overall results. In the proof-of-principle *Experiment A*, only one protein, TYMS, was identified (correctly) as a target candidate for 5-FU treatment. For PCTL, the list encompassed five proteins, including four tubulins (known targets). For TDX, two significant proteins were found, with the most probable candidate being TYMS (correct). The candidate list for MTX contains two proteins, with DHFR on the 1^st^ position (correct).

In the validation *Experiment B,* only TYMS was found (correctly) as a 5-FU target candidate. In the CAMP treatment, two proteins were shortlisted, including the known target TOP1. Unlike the behavior of other targets, TOP1 was significantly down-regulated upon CAMP treatment. Therefore, even in this limited experiment with only two cell lines, the correct results were obtained, with the lists of drug target candidates limited to one or two proteins.

### Mechanism of action

**Figure 3:**
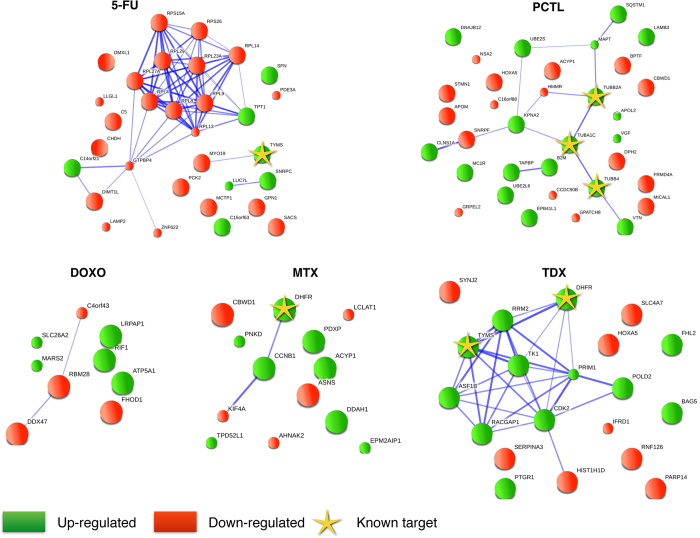
Protein-protein interaction networks obtained with STRING, for proteins with rank-product p ≤ 0.05 according to *Reg* and *Spec* rankings for (**a**) 5-FU, (**b**) TDX, (**c**) MTX, (**d**) PCTL and (e) DOXO treatments.

To highlight drug’s MoA, proteins sorted by their p-values derived from *Reg* and *Spec* rankings, with a cut-off at p ≤ 0.05, were mapped onto protein-protein interaction networks using STRING^16^. Exceptionality was not used in this case, because most of the significantly regulated proteins were downstream of the drug targets, and their regulation was not exceptional. In the proof-of-principle experiment, the p-value ranking gave 32 significant proteins for 5FU, 9 for DOXO, 13 for MTX, 34 for PCTL and 20 for TDX. These molecules were mapped on protein networks. For 5-FU, the main obtained cluster involves ribosome (Fig. 3a), the proteins of which were found specifically downregulated This observation is in line with the previous finding that ribosome suppression is a significant element of the 5-FU action^15^,^17^. Networks for other drugs are shown in Fig. 3b–e. As would be expected for non-random clusters, a great majority of proteins in clusters with ≥3 molecules have a same-sign regulation (up or down). For the largest clusters, the same regulation have 13 out of 15 5-FU proteins (down); 9 out of 11 PCTL proteins (up), and 9 out of 10 TDX proteins (up). According to gene set enrichment analysis, cytosolic large ribosomal subunit (GO cellular component) proteins were overrepresented in the 5-FU network (p = 1.8E-4); protein polymerization (GO biological process; p = 4E-4) in PCTL treatment; and pyrimidine metabolism (KEGG; p = 6E-5) in TDX treatment. These revealed mechanisms fully agree with literature data^17^,^18^,^19^.

## Discussion

Just a few years ago, cross-comparison of three cells lines at the baseline to the depth of 5,000 proteins has been reported for the first time^20^. Rapid recent progress in proteomics instrumentation and software have led to a marked decrease in the duration of a typical proteomics experiment, enabling analysis of ≥5,000 proteins in the time frame of ≤2 h^21^. This opened a previously unexplored opportunity to apply cellular proteomics to dozens^9^, and in perspective – hundreds and thousands of proteomes^8^, enabling cross-comparison between different cell lines grown at different conditions. Such a rapid development unlocked the analytical power of the proteome cross-comparison, which created a basis for the current study.

The panel of tested drugs encompasses such diverse mechanisms as DNA and/or RNA synthesis inhibitors (5-FU and TDX), antifolate agents (MTX), tubulin-active antimitotic agents (PCTL), and TOP1 inhibitors (CAMP)^3^. The results of the current study are supportive of the hypothesis that the protein drug target exhibits exceptional (unexpectedly large) regulation, unlike other proteins that change their abundance in harmony with the abundances of their co-regulated proteins. It is worth investigating how general this feature is, on a much larger panel of drugs, and with a broader panel of cell lines. The FITExP method, if proven general, can significantly shorten drug target identification, which is one of the major bottlenecks in the drug discovery procedure. Its findings need however be verified by orthogonal techniques, such as binding assays. Even with this limitation, high-content proteomics has a chance of becoming an important tool in drug target discovery.

## Methods

### Cell culture and drug treatments

HCT116 and RKO (colon carcinoma), H1299 (lung cancer), and A375 (melanoma) cell lines were kindly provided by colleagues from Karolinska Institutet. The cells were cultured at 37 °C with 5% CO_2_ in high-glucose Dulbecco’s Modified Eagle’s Medium (DMEM, Thermo Fisher Scientific) supplemented with 10% fetal bovine serum (Gibco) and 1% penicillin/streptomycin (Gibco). The cells were treated for 24–96 h with six different drugs: 5-fluorouracil (5FU), raltitrexed or tomudex (TDX), methotrexate (MTX) (all - Sigma), as well as doxorubicin (DOXO), paclitaxel (PCTL), and camptothecin (CAMP) (all - Eurasia Drugs, China). Each type of cell was treated with a concentration causing death of 15–50% of cells after 48 h of treatment. Concentrations used in Experiment A are listed in Table 2.

In Experiment B, the concentrations were 10 μM for 5-FU, 0.5 μM for PCTL, 30 nM for DOXO, and 15 μM for CAMP.

All drugs were dissolved in 0.01% dimethyl sulfoxide (DMSO). As a negative control, cells were treated with 0.01% DMSO. The medium supplemented with the drug was replaced each 24 h by fresh medium, except for starved/senescent cells that were left in the same medium for 10 days.

### Protein extraction and digestion

The collected cells were suspended in lysis buffer (1 mln cells in 100 μL buffer). The buffer was prepared by dissolving 1 mg ProteaseMax (Promega) in 900 μL ammonium bicarbonate (50 mM) and 100 μL acetonitrile (ACN). ProteaseMax is a surfactant which not only solubilizes the proteins but enhances subsequent tryptic digestion of proteins as well. The samples were vortexed for 5 min and then heated in shaking thermomixer (Eppendorf) at 50 ^o^C for 30 min at 1400 rpm, followed by sonication for 30 min. The total protein concentration was measured using the BCA protein assay kit (Pierce) in accordance with the manufacturer’s protocol. The extracted proteins were reduced with 5.5 mM dithiothreitol (DTT), alkylated with 15 mM iodoacetamide (IAA), and digested with 1.2 μg modified sequencing grade trypsin (Promega) dissolved in 50 mM ammonium bicarbonate. After 14 h of tryptic digestion, the reaction was stopped with acetic acid to a final concentration of 5% and then heated to 56 ^o^C for 30 min at 500 rpm (200 g), followed by centrifugation for 7 min at 14,000 rpm (10,000 g) at room temperature. The samples were pre-cleaned in a C18 column Zip-tip (Millipore), and the eluted peptides was dried in a SpeedVac centrifugal evaporator. The dried peptides were dissolved in water containing 1% formic acid (Fluka) for LC-MS/MS analysis. The above described digestion protocol was performed using the Mass Prep Station Robotic Protein Handling System (Waters, Manchester, UK).

### LC-MS/MS experiment

Chromatographic separation of peptide mixtures was achieved using a 50 cm Easy nanoflow column (Thermo; Experiment A) or a 75 μm ID fused silica column packed in-house (Experiment B) to the length of 8 cm with a slurry of reverse-phase, fully end-capped Reprosil-Pur C18-AQ 3 μm resin in methanol. No technical replicates were made, as the results from them were found to be practically identical. The peptides (5 μg for each biological replicate) were loaded onto the column at a flow rate of 1000 nL/min provided by nanoEasy UPLC (Thermo), and then eluted at a 300 nL/min flow rate for 180-210 min at a linear or biphasic gradient from 4% to 35% ACN in 0.1% formic acid. Electrospray ionization of the peptides was at 1.5 kV. The MS and MS/MS data was acquired in the Orbitrap mass analyzer (Orbitrap Q Exactive in Experiment 1 and Orbitrap Velos in Experiment 2) in the data-dependent acquisition mode. The survey MS spectrum was acquired at the resolution of 60,000 in the range of m/z 200 − 2000. MS/MS data were obtained with a higher-energy collisional dissociation (HCD) for ions with charge z≥2 at a resolution of 7,500 (Orbitrap Velos) or 15,000 (Q Exactive).

### Data processing

The raw files were converted to Mascot Generic Format (mgf) using in-house written Raw2mgf program. All mgf files were merged to create a common mgf file using in-house written Cluster program, which merged individual MS/MS spectra sharing more than 12 out of 20 most abundant peaks. The clustered mgf files were searched by the MS/MS search engine Mascot (version 2.3.0, Matrix Science, UK) to identify peptides and proteins. The mass tolerance was 10 ppm for precursor ions and 20 mDa for fragment ions, using carbamidomethyl (C) as a fixed modification, oxidation (M) as a variable modification, and up to two missed tryptic cleavages. The IPI human database (version 3.68; 91,521 human protein sequences) was searched, with reversed protein sequences concatenated as a decoy for determining the false discovery rate (FDR).

Quantitative information was extracted using in-house developed label-free software Quanti v.2.5.3.1^22^. Only reliably identified (FDR<0.01), unmodified peptides with unique sequences were considered and only proteins discovered with at least two such peptides were quantified. For each protein, one database identifier (ID) was selected, covering all the peptide sequences identified for this specific protein. If two proteins belonging to different protein groups had a partial sequence overlap, then all the peptides belonging to this overlap were ignored. The results were reported as a set of relative protein abundances ***A*** scaled such that the geometric mean of the abundance of each protein over all samples was 1.0.

### Scoring system

For combining the data from replicate analysis, “medians of ratios” are used instead of “ratios of medians”, as has previously been suggested^23^. If relative protein abundance of *i*-th quantified protein in *c*-th cell line under *j*-th treatment is denoted as 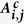, then regulat*i*on ***Reg*** is calculated as:


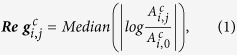


and specificity ***Spec*** is defined as:


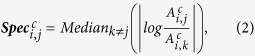


where j = 0 corresponds to untreated cells for ***Reg*** calculation, and *j≠k* for ***Spec*** calculations.

### Exceptional behavior measure

For each *I*-th protein and each *J*-th drug treatment, two vectors were calculated:


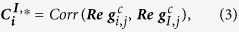



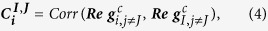


where 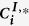 are the Pearson’s correlation coefficients of expression profiles over all treatments of *i*-th and *I*-th proteins, while 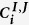 are correlation coefficients of the expression profiles of *i*-th and *I*-th proteins excluding treatment J. Then, the linear model 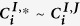 was created and the coefficient of determination of the model was used to calculate the measure of exceptional behavior *Exc*^*I,J*^ of *I*-th protein under *J*-th treatment:


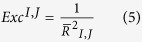


### p-value calculation

In estimation of the p-value of a protein with a certain rank, we used the rank product method, which has previously been found to be robust and tolerant to missing values in detection differentially regulated genes in replicated experiments^24^. The method has also been successfully applied to proteomics datasets for detection of significantly regulated proteins^25^. In adaptation of the method by Schwämmle et al., we treated ***Reg, Spec*** and ***Exc*** ranks as independent variables, and their values for different cell lines as well as at different incubation times were considered as independent replicate measurements. The rank product was considered to have a gamma distribution under null hypothesis, from which we calculated the p-values for the set of ranks of every protein. Adjusted p-values were calculated using standard Bonferroni correction, using the total number of proteins as a multiplication factor.

### Network mapping

STRING v9.1^16^ was used to map drug-specific, significantly regulated proteins onto protein-protein interaction networks. Gene names corresponding to up- and down-regulated proteins were submitted into STRING web-site (http://string-db.org). Medium confidence threshold (0.4) was used to define protein-protein interactions. Gene set enrichment analysis built in STRING with the whole genome background was used to identify enriched gene ontology terms and KEGG pathways. A 0.05% threshold was applied to the p-values after Benjamini-Hochberg correction.

### Availability of data and materials

Mass-spectra (Thermo raw files) were uploaded to chorus (chorusproject.org). Experimental details and data analysis algorithms, including the R program, as well as Excel files containing the data, are provided in Methods and Supplementary materials.

## Additional Information

**How to cite this article**: Chernobrovkin, A. *et al.* Functional Identification of Target by Expression Proteomics (FITExP) reveals protein targets and highlights mechanisms of action of small molecule drugs. *Sci. Rep.* **5**, 11176; doi:10.1038/srep11176 (2015).

## Supplementary Material

Supplementary Information

Supplementary Table (Dataset) 1

Supplementary Table (Dataset) 2

Supplementary Table (Dataset) 3

## Figures and Tables

**Table 1:** The drug target candidates (all proteins with p ≤ 0.05 for rankings of *Reg*, *Spec* and *Exc*) in proof-of-principle *Experiments A* and validation *Experiment B*, their up/down regulation and p-values (with Bonferroni correction).

**Drug**	**Experiment**	**Protein**	**Up/Down**	**p-value**
5-FU	*Experiment A*	**TYMS**	Up	4.7·10^−2^
	*Experiment B*	**TYMS**	Up	5.0·10^−2^
MTX	*Experiment A*	**DHFR**	Up	3.8·10^−8^
		TPD52	Up	2.7·10^−3^
PCTL	*Experiment A*	**TUBB2A**	Up	1.1·10^−4^
		**TUBB3**	Up	1.6·10^−3^
		**TUBB5**	Up	2.9·10^−3^
		**TUBA1C**	Up	3.8·10^−2^
		UBE2S	Up	4.9·10^−2^
TDX	*Experiment A*	**TYMS**	Up	1.1·10^−4^
		PRIM1	Up	2.9·10^−2^
CAM	*Experiment B*	CASP12	Up	2.1·10^−3^
		**TOP1**	Down	2.6·10^−3^

The known and expected targets are shown in bold.

**Table 2:** Drug concentrations causing death of 15–50% of cells after 48 h of treatment used in the Experiment A.

**CELL LINE**	**5-FU**	**TDX**	**DOXO**	**PCTX**	**MTX**	**CONTROLS**
HCT 116	50 μM	100 nM	5 μM	100 nM	5 μM	0 h, 72 h, SEN
A375	10 μM	50 nM	100 nM	50 nM	100 nM	0 h, 72 h, SEN
H1299	50 μM	10 μM	15 μM	100 nM	5 μM	0 h, 72 h, SEN
