# Supplementary material for: Functional Identification of Target by Expression Proteomics (FITExP) reveals protein targets and highlights mechanisms of action of small molecule drugs
Source: Sci Rep. 2015 Jun 8;5:11176. doi: 10.1038/srep11176 (PMC4459150; doi:10.1038/srep11176)
Supplement: Supplementary Information [file srep11176-s1.pdf]

Functional Identification of Target by Expression Proteomics (FITExP) reveals protein targets and highlights mechanisms of action of small molecule drugs

Alexey Chernobrovkin,[1] Consuelo Marin Vicente,[1,2] Neus Visa[2]

*and Roman A. Zubarev[1] **

[1]Division of Physiological Chemistry I, Department of Medical Biochemistry and Biophysics, Karolinska Institutet, Scheelesväg 2, SE-17 177 Stockholm, Sweden

[2]Department of Molecular Biology, Stockholm University, Stockholm, Sweden

SUPPLEMENTARY MATERIALS

Supplementary Table (Dataset) 1. Label-free proteomics quantification data and protein drug target identification results based on Regulation and Specificity for Experiment 1. Cell lines HCT116, A375, and H1299 were treated with 5-FU, TDX, MTX, PCTL and DOXO, as well as DMSO (control), or left in unchanged media for senescence/starvation (SEN).

Supplementary Table (Dataset) 2. Label-free proteomics quantification data and protein drug target identification results based on Regulation and Specificity for Experiment 2. Cell lines RKO and A375 treated with DOXO, 5-FU, CAMP and PCTL, as well as DMSO (control), or left in unchanged media for senescence/starvation (SEN).

Supplementary Table (Dataset) 3. Drug target identification results based on drug target exceptional behavior for Experiment 1. Cell lines HCT116, A375, H1299 treated with 5-FU, TDX, MTX, PCTL and DOXO, as well as DMSO (control), or left in unchanged media for senescence/starvation (SEN).

Functional Identification of Target by Expression Proteomics

Alexey Chernobrovkin

January 26, 2015

Data import and preprocessing

Raw mass-spectrometry data were processed by Quanti workflow. Protein-level quantitative data were put into supplementary table 1. The code here represents data analysis workflow for FITExP method.

```
require(gdata)
# data can be imported from original supplementary file (but its pretty slow)
#data <- read.xls('Supplementary Table 1.xlsx', 'initial data', head=T)
# or from csv file
data <- read.csv('data.csv', head=T)

rownames(data) <- data$PROTEIN.ID
columns.na <- apply(data[,grepl('_',names(data))], 2, function(x){sum(is.na(x))})
bad.columns <- names(columns.na[columns.na>(mean(columns.na)+sd(columns.na))])

data <- data[,!(names(data)%in% bad.columns)]
names(data) <- sub('5.FU','5FU', names(data))
filemask <- '([^_.]+)_([^_.]+)(\\.\\d+)?'
drugs <- unique(
                sub(
                    filemask,
                    '\\2',
                    grep('_', names(data), value=T)
                    )
                )
cells <- unique(
                sub(
                    filemask,
                    '\\1',
                    grep('_', names(data), value=T)
                   )
                )
```

4168 proteins were identified and quantified with at least two unique peptides over all samples. Data on 3 cell lines (Melanoma, Lung, Colon) and 8 conditions (CT0, 5FU, DOXO, MTX, CT72, PCTL, SEN, TDX) was loaded.

To calculate ratio of protein abundances in different treatments *median of the ratios* approach was used. For instance, if A1, A2 and A3 are abundances of protein in condition A, and B1, B2, B3 in condition B, then ratio $r_{A/B}$ would be defined as $median(A1/B1, A1/B2, ..., A3/B3)$.

Regulation and Specificity

Regulation is defined as relative abundance of a protein upon treatment compared to the selected control. Specificity is a relative abundance of a protein upon treatment compared to other treatments (excluding

controls).

```
# Regulation
control <- '(CT72)'
for(cell in cells){
    for(drug in drugs){
        target <- sprintf("%s_%s(\\.\\d+)?$",cell,drug)
        if(cell == 'Lung'){
            others <- "Lung_CT\\d+(\\.\\d+)?"
        }else{
            others <- sprintf("%s_%s(\\.\\d+)?$", cell, control)
        }
        res <- median.ratio(
            data,
            target,
            others
        )
        if(sum(!is.na(res))>0){
            data[,sprintf("%s_%s.regulation", drug, cell)] <- res
        }
    }
}

# Specificity
for(cell in cells){
    for(drug in drugs){
        target <- sprintf("%s_%s(\\.\\d+)?$",cell,drug)
        other.drugs <- setdiff(drugs, drug)
        other.drugs <- setdiff(other.drugs, control)
        other.drugs <- setdiff(other.drugs,c('CT0'))
        others <- sprintf(
                        "%s_(%s)(\\.\\d+)?$",
                        cell,
                        paste(other.drugs, collapse='|')
                        )
        res <- median.ratio(
            data,
            target,
            others
        )
        if(sum(!is.na(res))>0){
            data[,sprintf("%s_%s.specificity", drug, cell)] <- res
        }
    }
}
```

Rank product method realization on regulation and specificity values (produces MOA p-values).

```
require(xtable)
```

```
## Loading required package: xtable
```

```
suffix <- '(specificity|regulation)'
for(drug in setdiff(drugs,c('CT0','CT72','SEN'))){
    tRPMAData<-log2(data[,grepl(paste(drug,suffix,sep='.*'),names(data))])
        if(is.data.frame(tRPMAData)){
            NumElements<-rowSums(is.finite(!is.na(tRPMAData)))
            NumReps <- max(unique(NumElements))
            RPMAownUp_pvalues<-RPMAownDown_pvalues<-NULL
          for (d in unique(NumElements)) {
            RPMAData<-tRPMAData[NumElements==d,]
            if(d>1 && length(as.matrix(RPMAData))>ncol(tRPMAData)) {
              RP.own<-0
              Rank<-NULL
              RankNAs<-0
              for (r in 1:NumReps) {
                Rank[[r]]<-rank(RPMAData[,r],na.last="keep")/(sum(!is.na(RPMAData[,r]))+1)
                names(Rank[[r]]) <-rownames(RPMAData)
                Rank[[r]][is.na(Rank[[r]])]<-1
                RP.own<-RP.own+log(Rank[[r]])
                RankNAs<-RankNAs+sum(Rank[[r]]>1)
              }
              RP.own<-exp(RP.own)
              RPownCorr<- -log(RP.own)
              RPMAownUp_pvalues<-c(RPMAownUp_pvalues,pgamma(RPownCorr,d))

              RP.own<-0
              Rank<-NULL
              RankNAs<-0
              for (r in 1:NumReps) {
                Rank[[r]]<-rank(-RPMAData[,r],na.last="keep")/(sum(!is.na(RPMAData[,r]))+1)
                names(Rank[[r]]) <-rownames(RPMAData)
                Rank[[r]][is.na(Rank[[r]])]<-1
                RP.own<-RP.own+log(Rank[[r]])
                RankNAs<-RankNAs+sum(Rank[[r]]>1)
              }
              RP.own<-exp(RP.own)
              RPownCorr<- -log(RP.own)
              RPMAownDown_pvalues<-c(RPMAownDown_pvalues,pgamma(RPownCorr,d))
            }
          }
            RPMAown_pvalues<-2*apply(cbind(RPMAownDown_pvalues,RPMAownUp_pvalues),1,min)
          drug.res <- data.frame(
            row.names=names(RPMAown_pvalues),
            p.value=RPMAown_pvalues,
            updown=ifelse(RPMAownDown_pvalues>RPMAownUp_pvalues, 'Up','Down'))

            drug.res <- merge(drug.res, data[,c('PROTEIN.ID','DESCRIPTION','PEPTIDES')], by=0, all=F)
            drug.res$p.value[drug.res$p.value>1] <- 1
            drug.res$p.adjust <- p.adjust(drug.res$p.value, method='bonferroni')
            drug.res.print <- drug.res[order(drug.res$p.value),
                                     c('PROTEIN.ID',
                                       'DESCRIPTION',
                                       'PEPTIDES',
                                       'updown',
```

```
                                     'p.value',
                                     'p.adjust')]
     drug.res.print <- drug.res.print[drug.res.print$p.adjust<0.01,]
     print(
       xtable(
         drug.res.print,
         caption=sprintf("Proteins with significant and specific changes in abundance upon tratmen
         display=c('d','s','s','d','s','e','e')),
       scalebox=0.7,
       comment=F,
       timestamp=NULL,
       include.rownames = F,
       type='latex')
     #write.csv(drug.res[order(drug.res$p.value),],file=paste('corrp2_',drug,'.csv',sep=''))}

   }
}
```

PROTEIN.ID	DESCRIPTION	PEPTIDES	updown	p.value	p.adjust
IPI00410026	Isoform 2 of Putative RNA-binding protein Luc7-like 1	2	Up	1.73e-07	7.21e-04
IPI00013890	Isoform 1 of 14-3-3 protein sigma	13	Up	4.06e-07	1.69e-03
IPI00967757	Uncharacterized protein	3	Down	5.37e-07	2.24e-03
IPI00221108	Thymidylate synthase	5	Up	8.58e-07	3.58e-03
IPI00168603	Choline dehydrogenase, mitochondrial	4	Down	8.85e-07	3.69e-03
IPI00795015	Isoform 1 of Sacsin	9	Down	9.27e-07	3.86e-03
IPI00032291	Complement C5	3	Down	9.52e-07	3.97e-03
IPI00056499	Zinc finger protein 622	5	Down	1.09e-06	4.53e-03
IPI00977661	RPL17-C18orf32 protein isoform 1	8	Down	1.17e-06	4.87e-03
IPI00550900	Translationally-controlled tumor protein	6	Up	1.26e-06	5.24e-03
IPI00967907	Uncharacterized protein	2	Down	1.40e-06	5.83e-03
IPI00419919	Ribosomal protein L29	3	Down	1.66e-06	6.94e-03
IPI00641788	U1 small nuclear ribonucleoprotein C	2	Up	1.82e-06	7.59e-03
IPI00797038	Isoform 1 of Phosphoenolpyruvate carboxykinase [GTP], mitochondrial	18	Down	1.93e-06	8.03e-03
IPI00012772	60S ribosomal protein L8	11	Down	2.09e-06	8.70e-03

Table 1: Proteins with significant and specific changes in abundance upon tratment with 5FU

PROTEIN.ID	DESCRIPTION	PEPTIDES	updown	p.value	p.adjust
IPI00032107	Sulfate transporter	8	Up	8.50e-09	3.54e-05
IPI00026848	Alpha-2-macroglobulin receptor-associated protein	14	Up	2.31e-07	9.62e-04
IPI00440493	ATP synthase subunit alpha, mitochondrial	2	Up	1.79e-06	7.44e-03
IPI00375454	Isoform 2 of Telomere-associated protein RIF1	4	Up	1.84e-06	7.68e-03

Table 2: Proteins with significant and specific changes in abundance upon tratment with DOXO

PROTEIN.ID	DESCRIPTION	PEPTIDES	updown	p.value	p.adjust
IPI00030357	Dihydrofolate reductase	4	Up	8.01e-17	3.34e-13
IPI01009731	Uncharacterized protein	3	Down	1.41e-09	5.88e-06
IPI00977565	Uncharacterized protein	4	Up	3.28e-08	1.37e-04
IPI00925572	asparagine synthetase [glutamine-hydrolyzing] isoform b	16	Down	6.89e-07	2.87e-03
IPI00025340	Pyridoxal phosphate phosphatase	4	Up	8.56e-07	3.57e-03
IPI00221117	Acylphosphatase-1	3	Up	2.13e-06	8.89e-03
IPI00001022	Isoform 2 of Probable hydrolase PNKD	2	Up	2.15e-06	8.97e-03

Table 3: Proteins with significant and specific changes in abundance upon tratment with MTX

Correlation-based exceptionality assesment

For each I-th protein and each J-th drug treatment, two vectors were calculated:

$$C_i^{I,*} = Corr(Reg_{i,j}^c, Reg_{I,j}^c), C_i^{I,J} = Corr(Reg_{i,j\neq J}^c, Reg_{I,j\neq})^c),$$

PROTEIN.ID	DESCRIPTION	PEPTIDES	updown	p.value	p.adjust
IPI00930715	cDNA, FLJ93005, highly similar to Homo sapiens tubulin, beta polypeptide (TUBB), mRNA	2	Up	7.19e-12	3.00e-08
IPI00013683	Tubulin beta-3 chain	6	Up	1.59e-10	6.64e-07
IPI01022164	48 kDa protein	8	Up	1.33e-09	5.54e-06
IPI00645213	Uncharacterized protein	3	Down	2.62e-09	1.09e-05
IPI00785110	Isoform 1 of Nucleosome-remodeling factor subunit BPTF	3	Down	6.42e-09	2.67e-05
IPI00289501	Neurosecretory protein VGF	8	Up	7.14e-09	2.97e-05
IPI00844515	Isoform 2 of Hyaluronan mediated motility receptor	4	Down	4.51e-08	1.88e-04
IPI00554560	Protein C16orf88	2	Down	4.69e-08	1.95e-04
IPI00217949	Ubiquitin-conjugating enzyme E2 S	3	Up	4.78e-08	1.99e-04
IPI00549666	GrpE protein homolog 2, mitochondrial	5	Down	7.55e-08	3.15e-04
IPI00002214	Importin subunit alpha-2	14	Up	8.73e-08	3.64e-04
IPI00299404	Laminin subunit beta-3	6	Up	1.62e-07	6.74e-04
IPI00942874	Isoform 2 of G patch domain-containing protein 8	2	Down	1.82e-07	7.57e-04
IPI00218343	Tubulin alpha-1C chain	5	Up	2.27e-07	9.46e-04
IPI00007089	Ribosome biogenesis protein NSA2 homolog	2	Down	2.88e-07	1.20e-03
IPI01011752	Isoform 2 of DnaJ homolog subfamily B member 12	2	Up	2.91e-07	1.21e-03
IPI00386520	Isoform 2 of Ubiquitin/ISG15-conjugating enzyme E2 L6	3	Up	5.59e-07	2.33e-03
IPI00645410	Isoform 2 of Band 4.1-like protein 1	3	Up	6.39e-07	2.67e-03
IPI00977858	HOXA5 protein (Fragment)	2	Down	1.02e-06	4.24e-03
IPI00737502	diphthamide biosynthesis protein 2 isoform b	2	Down	2.06e-06	8.57e-03
IPI00220175	Isoform Tau-E of Microtubule-associated protein tau	3	Up	2.30e-06	9.59e-03

Table 4: Proteins with significant and specific changes in abundance upon tratment with PCTL

PROTEIN.ID	DESCRIPTION	PEPTIDES	updown	p.value	p.adjust
IPI00221108	Thymidylate synthase	5	Up	1.31e-12	5.44e-09
IPI00217466	Histone H1.3	5	Down	1.15e-11	4.79e-08
IPI00030357	Dihydrofolate reductase	4	Up	4.24e-10	1.77e-06
IPI00299214	Thymidine kinase, cytosolic	6	Up	2.99e-08	1.25e-04
IPI00925408	Uncharacterized protein	2	Down	6.36e-08	2.65e-04
IPI00031681	Cyclin-dependent kinase 2	5	Up	2.77e-07	1.15e-03
IPI00745921	Isoform 1 of RING finger protein 126	3	Down	6.77e-07	2.82e-03
IPI00743342	Four and a half LIM domains protein 2	7	Up	7.18e-07	2.99e-03
IPI00927879	Uncharacterized protein	8	Down	7.56e-07	3.15e-03
IPI00793754	DNA primase	3	Up	1.20e-06	4.99e-03

Table 5: Proteins with significant and specific changes in abundance upon tratment with TDX

where $C_i^{I,*}$ are the Pearson's correlation coefficients of expression profiles over all treatments of i-th and I-th proteins, while $C_i^{I,J}$ are correlation coefficients of the expression profiles of i-th and I-th proteins excluding treatment J. Then, the linear model $C_i^{I,*}$ $C_i^{I,J}$ was created and the coefficient of determination of the model was used to calculate the measure of exceptional behavior $Exc^{I,J}$ of I-th protein under J-th treatment:

$$Exc^{I,J} = 1/R^2_{I,J}.$$

Because of a number of proteins calculations could take a while

```
if(file.exists('rdata_all.csv')){
  rdata <- read.csv('rdata_all.csv', head=T, row.names=1)
}else{
  rdata0 <- data[,grepl('.*regulation', names(data))]
  rdata0 <- rdata0[,!grepl('CT0', names(rdata0))]
  ones <- apply(rdata0, 1, function(x){sum(x==1)})
  rdata <- rdata0[ones<5,]
  rdata <- na.exclude(rdata)
  #rdata <- log2(rdata)
  drugs <- setdiff(drugs,'CT0')
  res <- data.frame(row.names=rownames(rdata))
  corspec <- function(data, drug){
    tt <- apply(data, 1, function(rrow){
      r1 <- rrow[!grepl(drug, names(data))]
      cor1  <- apply(data[,!grepl(drug, names(data))],1, function(x){cor(r1, x)})
```

```
      r2 <- rrow
      cor2 <- apply(data, 1, function(x){cor(r2,x)})
      corlm <- lm(cor1~cor2)
      rs <- summary(corlm)$adj.r.squared
      slope <- corlm$coefficients[[2]]
      intercept <- corlm$coefficients[[1]]
      c(rs,slope,intercept)
      }
      )
    data.frame(t(as.data.frame(tt)))
    }
  require(parallel)
  lcorspec <- function(data, drug,method='p'){
    mdata <- data
    listed <- as.list.data.frame(as.data.frame(t(mdata)))
    mclapply(listed, function(rrow){
      r1 <- rrow[!grepl(drug, names(mdata))]
      cor1  <- apply(mdata[,!grepl(drug, names(mdata))],1, function(x){cor(r1, x,method=method)})
      r2 <- rrow
      cor2 <- apply(mdata, 1, function(x){cor(r2,x,method=method)})
      corlm <- lm(cor1[cor1>0&cor2>0]~cor2[cor1>0&cor2>0])
      rs <- summary(corlm)$adj.r.squared
      slope <- corlm$coefficients[[2]]
      intercept <- corlm$coefficients[[1]]
      c(rs,slope,intercept)
      }, mc.cores=8
      ) -> tt
    data.frame(t(as.data.frame(tt)))

    }
  for(drug in setdiff(drugs, c('CT0','CT72'))){
    tmp <- data.frame()
    tmp <-  lcorspec(rdata, drug, 'p')
    names(tmp) <- paste(drug, c('adj.r.squared','slope','intercept'), sep='.')
    res <- cbind(res,tmp)
    }
  data$DESCRIPTION[match(rownames(rdata), data$PROTEIN.ID)] -> res$description
  write.csv(cbind(rdata,res), 'rdata_all.csv')
}
```

Selecting variables for rank product analysis

```
suffix <- '(regulation)'
rd2 <- merge(
             data[,grepl(suffix,names(data))],
             1/rdata[,grepl('(square)',names(rdata))],
             by=0
             )
rownames(rd2) <- rd2[,1]
rd2 <- rd2[,2:ncol(rd2)]
```

Rank product method

```
for(drug in setdiff(drugs,c('CT0','CT72'))){
  drug.res <- data.frame(row.names=rownames(rd2))
  tRPMAData<-log2(rd2[,grepl(drug,names(rd2))])
  if(is.data.frame(tRPMAData)){
    NumElements<-rowSums(is.finite(!is.na(tRPMAData)))
    NumReps <- max(unique(NumElements))
    RPMAownUp_pvalues<-RPMAownDown_pvalues<-NULL
    for (d in unique(NumElements)) {
      RPMAData<-tRPMAData[NumElements==d,]
      if(d>1 && length(as.matrix(RPMAData))>ncol(tRPMAData)) {
        RP.own<-0
        Rank<-NULL
        RankNAs<-0
        for (r in 1:NumReps) {
          Rank[[r]]<-rank(RPMAData[,r],na.last="keep")/(sum(!is.na(RPMAData[,r]))+1)
          names(Rank[[r]]) <-rownames(RPMAData)
          Rank[[r]][is.na(Rank[[r]])]<-1
          RP.own<-RP.own+log(Rank[[r]])
          RankNAs<-RankNAs+sum(Rank[[r]]>1)
          }
        RP.own<-exp(RP.own)
        RPownCorr<- -log(RP.own)
        RPMAownUp_pvalues<-c(RPMAownUp_pvalues,pgamma(RPownCorr,d))

        RP.own<-0
        Rank<-NULL
        RankNAs<-0
        for (r in 1:NumReps) {
          Rank[[r]]<-rank(-RPMAData[,r],na.last="keep")/(sum(!is.na(RPMAData[,r]))+1)
          names(Rank[[r]]) <-rownames(RPMAData)
          Rank[[r]][is.na(Rank[[r]])]<-1
          RP.own<-RP.own+log(Rank[[r]])
          RankNAs<-RankNAs+sum(Rank[[r]]>1)
          }
        RP.own<-exp(RP.own)
        RPownCorr<- -log(RP.own)
        RPMAownDown_pvalues<-c(RPMAownDown_pvalues,pgamma(RPownCorr,d))
        }
      }
    RPMAown_pvalues<-2*apply(cbind(RPMAownDown_pvalues,RPMAownUp_pvalues),1,min)
    drug.res <- data.frame(
      row.names=names(RPMAown_pvalues),
      p.value=RPMAown_pvalues,
      updown=ifelse(RPMAownDown_pvalues>RPMAownUp_pvalues, 'Up','Down'))

    drug.res <- merge(drug.res, data[,c('PROTEIN.ID','DESCRIPTION','PEPTIDES')], by=0, all=F)
    drug.res$p.value[drug.res$p.value>1] <- 1
    drug.res$p.adjust <- p.adjust(drug.res$p.value, method='bonferroni')
    drug.res.print <- drug.res[order(drug.res$p.value),
                               c('PROTEIN.ID',
                                 'DESCRIPTION',
                                 'PEPTIDES',
                                 'updown',
```

```
                                  'p.value',
                                  'p.adjust')]
    drug.res.print <- drug.res.print[drug.res.print$p.adjust<0.051,]
    print(
      xtable(
        drug.res.print,
        caption=sprintf("Drug target candidates for %s ", drug),
        display=c('d', 's','s','d','s','e','e')),
      type='latex',
      scalebox=0.7,
      comment=F,
      timestamp=NULL,
      include.rownames = F)
    #write.csv(drug.res[order(drug.res$p.value),],file=paste('corrp_',drug,'.csv',sep=''))}
  }
}
```

PROTEIN.ID	DESCRIPTION	PEPTIDES	updown	p.value	p.adjust
IPI00221108	Thymidylate synthase	5	Up	1.19e-05	4.69e-02

Table 6: Drug target candidates for 5FU

PROTEIN.ID	DESCRIPTION	PEPTIDES	updown	p.value	p.adjust
IPI00005401	Polypeptide N-acetylgalactosaminyltransferase 5	2	Up	1.77e-06	7.01e-03

Table 7: Drug target candidates for DOXO

PROTEIN.ID	DESCRIPTION	PEPTIDES	updown	p.value	p.adjust
IPI00030357	Dihydrofolate reductase	4	Up	9.71e-12	3.84e-08
IPI00977565	Uncharacterized protein	4	Up	6.94e-07	2.74e-03

Table 8: Drug target candidates for MTX

PROTEIN.ID	DESCRIPTION	PEPTIDES	updown	p.value	p.adjust
IPI00930715	cDNA, FLJ93005, highly similar to Homo sapiens tubulin, beta polypeptide (TUBB), mRNA	2	Up	2.89e-08	1.14e-04
IPI00013683	Tubulin beta-3 chain	6	Up	3.94e-07	1.56e-03
IPI01022164	48 kDa protein	8	Up	7.22e-07	2.86e-03
IPI00218343	Tubulin alpha-1C chain	5	Up	9.64e-06	3.81e-02
IPI00217949	Ubiquitin-conjugating enzyme E2 S	3	Up	1.23e-05	4.87e-02

Table 9: Drug target candidates for PCTL

PROTEIN.ID	DESCRIPTION	PEPTIDES	updown	p.value	p.adjust
IPI00888907	Isoform 2 of Cyclin-D1-binding protein 1	2	Up	8.55e-08	3.38e-04
IPI00645038	Uncharacterized protein	6	Up	1.61e-07	6.38e-04
IPI00413641	Aldose reductase	16	Up	1.96e-07	7.77e-04
IPI01010566	Zinc finger and BTB domain-containing protein 38	3	Up	1.81e-06	7.18e-03
IPI00878498	35 kDa protein	2	Up	4.17e-06	1.65e-02
IPI00871221	Isoform 2 of Sodium/potassium-transporting ATPase subunit beta-1	9	Up	4.73e-06	1.87e-02
IPI00916849	Protein	2	Up	6.31e-06	2.50e-02

Table 10: Drug target candidates for SEN

PROTEIN.ID	DESCRIPTION	PEPTIDES	updown	p.value	p.adjust
IPI00221108	Thymidylate synthase	5	Up	2.66e-08	1.05e-04
IPI00793754	DNA primase	3	Up	7.45e-06	2.95e-02

Table 11: Drug target candidates for TDX
